# Network Pharmacology Studies on the Bioactive Compounds and Action Mechanisms of Natural Products for the Treatment of Diabetes Mellitus: A Review

**DOI:** 10.3389/fphar.2017.00074

**Published:** 2017-02-23

**Authors:** Weiwei Li, Guoqi Yuan, Yuxiang Pan, Cong Wang, Haixia Chen

**Affiliations:** Tianjin Key Laboratory for Modern Drug Delivery & High-Efficiency, School of Pharmaceutical Science and Technology, Tianjin UniversityTianjin, China

**Keywords:** network pharmacology, diabetes mellitus, natural products, database, mechanism

## Abstract

Diabetes mellitus (DM) is a kind of chronic and metabolic disease, which can cause a number of diseases and severe complications. Network pharmacology approach is introduced to study DM, which can combine the drugs, target proteins and disease and form drug-target-disease networks. Network pharmacology has been widely used in the studies of the bioactive compounds and action mechanisms of natural products for the treatment of DM due to the multi-components, multi-targets, and lower side effects. This review provides a balanced and comprehensive summary on network pharmacology from current studies, highlighting different bioactive constituents, related databases and applications in the investigations on the treatment of DM especially type 2. The mechanisms related to type 2 DM, including α-amylase and α-glucosidase inhibitory, targeting β cell dysfunction, AMPK signal pathway and PI3K/Akt signal pathway are summarized and critiqued. It suggests that the network pharmacology approach cannot only provide a new research paradigm for natural products, but also improve the current antidiabetic drug discovery strategies. Furthermore, we put forward the perspectives on the reasonable applications of network pharmacology for the therapy of DM and related drug discovery.

## Introduction

Diabetes mellitus (DM) has drawn much attention of researchers due to its increasing mortality and complex complications. According to the International Diabetes Federation, DM has become a major threat to the health and the third biggest killer after cardiovascular diseases and cancer^1^. Now DM has terrible influences on both high- and low-income countries, with bearing the majority of the burden in low-income countries. According to the latest statistics of WHO, DM directly caused 1.5 million deaths in 2012. There were approximately 422 million (1 in every 11 people) diabetics worldwide in 2014, which was 14-fold more than those in 1980^2^. And the cases will reach 600 million by 2030 based on the developing trend (Kokil et al., 2015).

Diabetes mellitus is a group of chronic and metabolic diseases, in which there are high blood glucose levels over a long period. It is caused by either the body can’t produce enough insulin or the body can’t effectively respond to the produced insulin. Based on different pathogenesis, DM can be categorized into three types. Type 1 diabetes mellitus (T1DM), namely insulin-dependent DM or childhood-onset diabetes, is caused by insulin deficiency, which is related to dysfunction β cells (Enk and Mandelboim, 2014). Type 2 diabetes mellitus (T2DM), namely non-insulin-dependent DM or adult-onset diabetes, is characterized by insulin resistance (IR) and relatively insulin secretion reducing. There is another type of the diabetes, gestational diabetes mellitus (GDM), which is characterized by high blood glucose levels in pregnant women without previously diagnosed diabetes (Keller et al., 2008; Puri and Hebrok, 2012). According to clinical and non-clinical studies, T2DM is the main category accounting for about 90% of diabetes cases, which is also the focus of this review. The risk factors for T2DM are relatively clear including age, obesity, lifestyle, dietary patterns gene-environment interactions and epigenomics, etc. (Herman and Zimmet, 2012; Karter et al., 2013; Maruthur, 2013). It is reported that multiple genes are involved in genetic susceptibility. The pathogenesis of the DM diseases are closely related to the alterations in multiple signal pathways such as JAK-STAT, AMPK, and PI3K (Richard and Stephens, 2011; Samuel and Shulman, 2012). And the changes of microbial environment might affect the DM pathogenesis (Wen et al., 2008). Study of Qin showed that gut microbial dysbiosis and pathogenic bacteria increasing were observed in patients with T2DM (Qin et al., 2012). If left untreated, DM can cause many complications, including kidneys damage, eyes damage, heart disease, stroke, bone fracture risks, and function failure (Jason et al., 2016). So many recent studies are focusing on the anti-diabetic drugs discovery.

A series of medicines, including analogs, sulfonylureas, thiazolidinedione, biguanides, α-glucosidase inhibitors, and DDP-4 inhibitors, have been used as commercial products for therapy of DM (Morral, 2003; Tian N. et al., 2013). However, because of the “one target, one drug” mechanism, they might have similar side effects, such as weight gain, cardiovascular disease, glycopenia, and gastrointestinal effects (Shah and Mudaliar, 2010). Considering the long-term treatment, side effects and high price of the current antidiabetic drugs, there is a huge demand for effective, low-toxic or non-toxic and affordable drugs for DM.

Compared with the synthetic drugs, natural products have played an important role in DM treatment. The treatment benefits are attributed to multi-components and multi-targets to produce combined or synergistic effects. With the rapid development of novel technologies, increasing studies have been focused on antidiabetic natural products. Nowadays, multi-components and multi-targets therapies have been proved to be more effective and less toxicity than traditional single-target drugs (Espinoza-Fonseca, 2006). With the rapid progress of bioinformatics, network pharmacology, integrating network biology and pharmacology, is considered as a promising approach toward more cost-effective drug development (Khan and Khan, 2016). It can reveal the underlying complex relationship between multi-components and multi-targets. Moreover, network pharmacology provides a system-level approach to understand the pathogenesis of diseases, and can be used for lead discovery, target identification and indication prediction, which coincides with the holistic and systematic concepts in traditional Chinese medicine (TCM) theory (Hopkins, 2008; Tang and Aittokallio, 2014). Compared with conventional “one gene, one drug, one disease” mode, network pharmacology focuses on “multi-targets, multi-components” treatment to diseases. And signal network analysis is a highly attractive tool to investigate complex relationships. What’s more, based on network pharmacology, feasible “drug-target-disease” network models can be established through high-throughput screening and bioinformatics (Poornima et al., 2016). It was reported that network pharmacology had various applications, such as herbal ingredient target/disease gene prediction, network balance regulation, elucidation of synergistic ingredient pairs and active ingredient groups (Tao et al., 2013; Zhang X. et al., 2014; Zintzaras et al., 2014). Thus, strategies based on network including network pharmacology and network medicine are increasingly developed and applied. And several databases have been established to assist network pharmacology analysis including PhIN (pharmacology interaction network database) (Wang Z. et al., 2015), C^2^Maps (computational connectivity maps) (Huang et al., 2012), databases on TCM (Li and Zhang, 2013), CVDHD (The cardiovascular disease herbal database) (Gu et al., 2013b) and anticancer drugs database (Azmi et al., 2010).

In drug discovery, combined multi-components and multi-targets, natural products based on network pharmacology can afford bright perspectives for treating DM in a systematic manner. It is an efficient and time-saving method to find the potential application in other therapeutic categories of drugs by predicting their targets. Therefore, the network pharmacology approach is developed to find effective drugs for the treatment of DM and related metabolic disorders such as obesity and metabolic syndrome. Based on network pharmacology, the antidiabetic compounds were detected from Ge-Gen-Qin-Lian decoction formula, and the mechanisms were elucidated (Li et al., 2014). In the study of Zhang S. et al. (2014), the “drug-target-pathway-disease” network of green tea was established. And neurotrophin signal pathway, p53 signal pathway, and T2DM pathway related to DM were screened by network pharmacology (Zhang S. et al., 2014). It was reported that the network pharmacology analysis was developed to illustrate the action mechanism of Tangminling Pills for T2DM therapy (Gu et al., 2011).

The aim of this review is to provide a summary of network pharmacology studies on the bioactive compounds and action mechanisms of natural products for the treatment of DM. The recent progresses on the different active constituents, databases and applications in the treatment of DM were highlighted. The mechanisms related to T2DM, including α-amylase and α-glucosidase inhibitory, targeting β cell dysfunction, AMPK signal pathway, PI3K/Akt signal pathway and modulation of gut microbiota were summarized. And the perspectives on their reasonable applications for therapy of DM and related drug discovery were put forward.

## Network Pharmacology on DM

### Databases of Natural Products

Natural products have played significant roles in the leads for drug discovery, which were involved in the development of approximately 64% of all drugs (Newman and Cragg, 2012). Databases of natural products are important for screening in drug discovery. Thus, several databases were established to provide the related information of natural products.

Super Natural II^3^ (Banerjee et al., 2015), containing about 326,000 molecules, was a freely available database to find natural products with different incorporating search functions. The database contained approximately 170,000 compounds and provided the following information of the products, including two-dimentional structures, the corresponding structural and physicochemical properties, the predicted toxicity class and the vendor information etc.

NAPRALERT (Loub et al., 1985) was a source database. The database collected the information of natural products by textual-numeric. About 105,000 organism names, 195,000 pharmacological results and 190,000 identified compounds were collected. In addition, it provided a strategy to prophetically identify the classification source of the most promising specific biological activities.

Chemical Entities of Biological Interest (ChEBI)^4^ (And et al., 2003) was also a freely available dictionary focused on small chemical compounds of either natural products or synthetic products. The database represented more than 12, 000 molecular entities, groups and classes. Some important information was provided such as ChEBI ID, ChEBI names, definition, structural diagrams, formula, synonyms, and registry number.

DrugBank (Wishart et al., 2008) was a database enriched drug data. The 2.0 version of DrugBank presented about 4,900 drug entries, which included about 1,467 FDA-approved drugs. In addition, the database contained common links to nearly all primary bioinformatics and biomedical databases (GenBank, KEGG, PubChem, and PubMed).

Other databases related to natural products were also developed, such as PubChem (Wang et al., 2009), UPND (Universal Natural Products Database) (Gu et al., 2013a), Dictionary of Natural Products (DNPs)^5^, CHDD (Qiao et al., 2002), Herb BioMap database (China Copyright of Computer Sofware, 2011SR076502), Chinese Natural Product Database (CNPD) (Zheng et al., 2005), Traditional Chinese Medicines Database (TCMD) (He et al., 2001) and ChemBank (Seiler et al., 2008).

All the databases can be useful for the studies involving virtual screening and the design of new compounds from natural compounds. The information about structures and physicochemical properties is useful and it can contribute to future drug development.

### Natural Products for DM Therapy

Natural products have played an important role in DM therapy for a long history, especially in Asia, India and Africa. There were massive studies focused on herbal medicines for the development and discovery of antidiabetic medicine. Many kinds of extracts and bioactive constituents were studied on the hypoglycemic effects.

#### Extracts

Extracts from natural products have been widely prescribed for diabetic treatment worldwide. Some of them were evaluated scientifically and methodically in order to check for their properties (Odhav et al., 2010). Various plant extracts were traditionally used to DM treatment. In a long-term study, *Annona muricata* aqueous extract was daily administrated to diabetic rats for 28 days, the blood glucose levels and serum creatinine were reduced, and the MDA, AST, ALT activity, and nitrite levels LDL-cholesterol were also reduced (Florence et al., 2014). Khanra et al. (2015) reported that *Abroma augusta L.* (*Malvaceae*) leaf extract could be considered as a kind of prophylactic agent against T2DM and its associated kidney and cardiac toxicity (Khanra et al., 2015). Chicory seed extract had the capacity to target hyperglycemia, hyperlipidemia, IR, NAFLD (non-alcoholic fatty liver disease) and NASH (non-alcoholic steatohepatitis) through modulating PPARα/SREBP-1 ratio (Ziamajidi et al., 2013). There were other extracts used for the DM treatment, such as *Hypericum perforatum* extract (Hasanein and Shahidi, 2011), grape seed and skin extract (Oueslati et al., 2016), and flavonoid rich extract from *Sophora tonkinensis* Gagnep (Huang et al., 2016). So the herbal extracts can be used for the treatment of T2DM at systematic levels.

#### Polysaccharides

Polysaccharides are one of the main components of the natural sources and they are composed of more than 10 monosaccharide units linked together by glycosidic bonds. As the main bioactive fractions of natural products, polysaccharides have attracted much more attention during recent years (Zong et al., 2012). As shown in **Table 1**, polysaccharides from natural products were applied for the DM treatment during the period 2010–2016.

**Table 1:** Polysaccharides applied on the treatment of diabetes mellitus.

Class	Origin	Effect and mechanisms	Reference
Plant	*Astragalus membranaceus*	Improving insulin sensitivity; decreasing myostatin expression; downregulating ROS-ERK-NF-κB pathway	Liu et al., 2013
	*Liriope spicata*	Improving PI3K signal pathway; upregulating the protein expression of PPARγ; improving glucose metabolism	Xiao et al., 2014
	*Lycium barbarum*	Delaying the absorption of glucose; reducing the postprandial blood glucose	Tang et al., 2015
	*Ophiopogon japonucus*	Regulating InsR/IRS-1/PI3K/Akt/GSK-3/Glut-4 signal pathway	Wang et al., 2012
	*Cucurbita moschata*	Decreasing the levels of TG, TC, and LDL, cholestrol; increasing the levels of fecal fat, and HDL	Zhao et al., 2013
	*Panax ginseng*	Alleviating oxidative stress; stimulating increased insulin secretion	Sun et al., 2014
Mushroom	*Ganoderma Lucidum*	Upregulating Bcl-2 and PDX-1; downregulating Bax, iNOS, and Casp-3 mRNA expressions	Zheng et al., 2012
	*Ganoderma atrum*	Activating PI3K/Akt/Enos signal pathway	Zhu et al., 2014
	*Grifola frondosa*	Increasing the metabolism of glucose; regulating Akt/GSK-3 signal pathway	Ma et al., 2014
Seaweed	*Enteromorpha prolifera*	Regulating the mRNA level of InsR, GCK, APN, and GLUT-4 gene in liver and adipose tissue	Lin et al., 2015
Bacterial	*Trametes gibbosa*	Decreasing the levels of LDL-C BG, TG, and TC; increasing the level of HDL-C	Ma et al., 2013
Animal	*Misgurnus anguillicaudatus*	Elevating the insulin level; increasing PEPCK mRNA expression; reducing glycogen contents	Zhou et al., 2015

According to our previous study (2015), tea polysaccharides (TPS) were found to increase the body weight and decrease the blood glucose. It had higher potent glucose tolerance. And some biochemical indices were ameliorated. The TC and LDL-c contents were decreased and the TG and HDL-c contents were restored nearly to the normal levels. Furthermore, via upregulating the expressions of PI3K, p-Akt and GLUT4 target proteins in PI3K/Akt signal pathway, TPS could play an effective role in hypoglycemia. It was indicated that TPS might be a promising candidate for T2DM treatment (Li et al., 2015). A water soluble TPS enhanced insulin secretion simulated by glucose through cAMP-PKA pathway by upregulating not only the mRNA transcription but also the expression of PDX-1 protein (Wang H. et al., 2015). Polysaccharides of corn silk (POCS) were evaluated for its antidiabetic effects on streptozotocin (STZ)-induced diabetic rats. The results showed that POCS could not only significantly decrease blood glucose level, but also reduce total cholesterol (TC) and total triglyceride (TG) (Zhao W. et al., 2012). Mulberry leaf polysaccharides were reported to effectively improve the hepatic glucose metabolism and IR to the normal levels. By inhibiting the expression of protein–tyrosine phosphatase 1B, activating the PI3K/AKT signal pathway and relieving oxidative stress, the polysaccharides were proved to be a prophylactic agent for T2DM (Ren et al., 2014). What’s more, polysaccharides from *Lachnum calyculiforme* (Ye et al., 2011) and polysaccharides from *Cynomorium songaricum* (Wang et al., 2010) exhibited obvious hypoglycemic effects. The polysaccharides can be applied in the treatment of DM by targeting different signal pathways.

#### Polyphenols

Antidiabetic polyphenols extracted from natural products in the period 2010–2016 were listed in **Table 2**. Polyphenols, the secondary metabolites of plants, are mainly responsible for the flavor and color of fruits and other plant products. They are presented in fresh fruits and vegetables and found in various natural beverages such as red wines, tea and cocoa (Quideau et al., 2011). Solayman et al. (2015) reviewed the categories of polyphenols in the DM treatment, including anthocyanin, ellagitannin, luteolin, rosmarinic acids, catechin, resberatrol, rutin, quercetin, diosimin, and myricetin (Solayman et al., 2015). Different polyphenols have different action mechanisms.

**Table 2:** Polyphenols applied on the treatment of diabetes mellitus.

Origin	Effects and mechanisms	Reference
*Ecklonia cava*	Improving blood glucose regulation; regulating hepatic glucose metabolic; upregulating Akt protein	Kim et al., 2016
*Folium Mori*	Increasing the mRNA and protein expression of IRS-1 of PI3K-p85α and Glut-4IRS-1/PI3K/Glut-4 signal pathway	Cai et al., 2016
*Litchi chinensis Sonn.*	Improving glucose tolerance and insulin resistance; influencing lipid metabolism; increasing mRNA levels of Bax and NF-κB	Man et al., 2016
*Corchorus olitorius*	α-amylase and α-glucosidase inhibitory; high antioxidant capacity	Oboh et al., 2012
*Theobroma cacao L.*	Ameliorating insulin sensitivity, glucose uptake, and adiponectin secretion via regulating MAPK signal pathway	Ali et al., 2014
*Grape seed*	Regulating MFG-E8, IL-1β and NLRP3	Yin et al., 2015
*Curcuma longa*	Inhibiting the activation of the SphK1-S1P signal pathway	Huang et al., 2013

It was reported that polyphenols from black soybean seed coat improved hyperglycemia and insulin sensitivity via regulating AMPK signal pathway both *in vitro* and *in vivo*, and anthocyanins [cyanidin 3-glucoside (C3G)] and procyanidins (PCs) were the main antidiabetic polyphenols, which could also enhanced glucose uptake (Kurimoto et al., 2013). Polyphenols from *Vernonia amygdalina* were reported to possess antihyperglycemic effects, most probably via increasing GLUT4 translocation and inhibiting hepatic G6Pase (Ong et al., 2010). EGCG, a kind of green tea polyphenol, could improve endothelial dysfunction and ameliorate metabolic IR in skeletal muscle and liver. And it was reported that EGCG could attenuate the death of β-cells in the db/db mouse, reduce IR, and increase the glucose-induced insulin scretion (Ortsäter et al., 2012). Therefore, the polyphenols could be a promising therapy to treat T2DM, obesity, and cardiovascular, in which there were reciprocal relationships between IR and endothelial dysfunction (Keske et al., 2015).

#### Other Constituents

Besides polysaccharides and polyphenols, there are other constituents from natural products related to antidiabetic effects. Several reviews provided summaries of the natural constituents for DM therapy, such as terpenoids, tannins, saponins, alkaloids, and lignans (Tundis et al., 2010; Hung et al., 2012; Zhang and Jiang, 2012).

Traditional herbal medicine has been used to treat DM for decades. Recent studies of antidiabetic compounds from 2010 to 2016 were categorized here. According to structure-activity relationship, these action mechanisms of natural products are quite different from those of the currently antidiabetic drugs. Natural products might be the promising sources to provide multi-components with multi-targets and new therapy for DM.

### Database of DM

Network pharmacology is a new strategy to find the bioactive constituents as well as the molecular targets and the interactions. Nowadays, there are more studies and knowledge of DM from published literatures and the protein–protein interaction (PPI) databases (Liechti et al., 2010). Several databases such as T1Dbase, T2D-Db, T2DGADB, and T2D@ZJU have integrated the existing information, which contained genetic association studies, gene expression, related signal pathways, and PPI.

T1Dbase was a public website and database related to the molecular genetics and biology of T1DM. T1Dbase integrated the data from assembled genome sequences, derived gene and transcript models, publications linked to genes, T1DM susceptibility regions, genetic linkage and related studies. It provided integrated data and opportunities for researchers to explore the complex pathogenesis of T1DM which was affected by various factors (Hulbert et al., 2007).

T2D-Db collected 330 candidate genes from the Pubmed literatures and provided their corresponding information on almost all known molecular components involved in the pathogenesis of T2DM. Information on candidate genes had been established in this on line database, including SNPs (single nucleotide polymorphism) in candidate genes or candidate regions, GWA (genome wide association studies), pathways, PPI and diseases associated risk factors or complications (Agrawal et al., 2008).

T2DGADB collected 701 publications in T2DM genetic associated research area. It aimed to provide specialized information on the genetic risk factors involved in the development of T2DM. And T2DGADB focused on information related to each SNP association including the populations used, odds ratio and other factors (Lim et al., 2010).

T2D@ZJU summarized three levels of heterogeneous connections related to T2DM, which was searched from pathway databases, PPI databases and literatures. It contained 1,078 T2DM related substances including proteins and its complexes, drugs and the interactions. Compared with T2D-Db and T2DGADB, which focused on the genes associated with T2DM, T2D@ZJU organized the integrated information through network. From the perspective of system biology, it established a basis for the further research of T2DM, which was contributed to the elucidation of the action mechanism and related drug development. Furthermore, this database could also promote the T2DM-related studies of network pharmacology and multi-targets therapeutics (Yang et al., 2013).

The high availability of the DM databases plays a significant role in integrating related complex data. They can be used to predict the target network of the bioactive constituents. After experimental validation of the molecular network, they can provide a new perspective for researchers to better understand the pathogenesis of DM and action mechanisms of associated drugs.

### Action Mechanisms Related to T2DM

A large amount of studies have indicated that natural products showed antidiabetic effects via a variety of mechanisms. The present review summarizes and discusses the related antidiabetic mechanisms.

#### α-Amylase and α-Glucosidase Inhibitory

It has been reported that the inhibition of α-amylase and α-glucosidase could be an important concept for therapy of T2DM. Dietary carbohydrates can be hydrolyzed into oligosaccharide and then into monosaccharide including glucose, which is produced by the main hydrolyzing enzymes such as pancreatic α-amylase and intestinal α-glucosidase. The α-amylase hydrolyzes α-1,4-glycocidic bonds and produced smaller oligosaccharides and disaccharides, while the α-glucosidase hydrolyzes disaccharides to monosaccharide (Apostolidis and Lee, 2010). Therefore, it is an effective approach to manage the blood glucose level via inhibiting the activities of α-amylase and α-glucosidase (Striegel et al., 2015).

#### Targeting β Cell Dysfunction

The β cell dysfunction and IR are inherently complex with their interrelation for triggering the pathogenesis of DM. It results from inadequate glucose sensing to stimulate insulin secretion. Cytokine-induced inflammation, oxidative stress, and overconsumption of saturated fat and free fatty acids can cause β cell dysfunction (Cerf, 2013). Therefore, preserving β cell function with anti-inflammatory effect and antioxidant effect can maintain glucose homeostasis (Nguyen et al., 2012; Hasnain et al., 2016).

#### Targeting Signal Pathways

**FIGURE 1:**
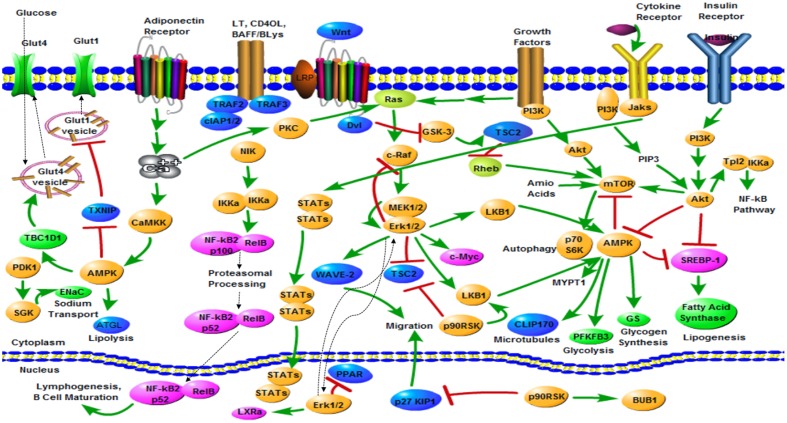
**Summary of signal pathways related to diabetes mellitus**. Pathway diagram keys: 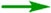 Direct stimulatory modification; 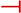 Direct inhibitory modification; 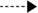 Translocation; 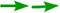 Multistep stimulatory modification.

There are various sophisticated signal pathways related to DM that can be therapeutically targeted. The present review provides a summary of signal pathways related to DM (**Figure 1**), including AMPK signal pathway (Kurimoto et al., 2013), PI3K/Akt signal pathway (Li et al., 2015), mTOR signal pathway (Siegel, 2008), JAK-STAT signal pathway (Gurzov et al., 2016), ROS-ERK-NF-κB signal pathway (Liu et al., 2013), Wnt signal pathway (Chiang et al., 2012), and IGF-1 signal pathway (Siddle, 2011). By targeting these signal pathways, the bioactive constituents can produce multiple effects including glut translocation, glut trafficking, glycogen synthesis, glycolysis, lipolysis, microtubules, gluconeogenesis, lipogenesis, and autophagy.

#### Modulation of Gut Microbiota

The gut microbiota was hypothesized to play a critical role in metabolic diseases, including T2DM (Xu et al., 2015). Ge-Gen-Qin-Lian decoction (GQD) formula, consisted of four herbs: *Puerariae Lobatae radix* (Ge-Gen), *Coptidis rhizoma* (Huang-Lian), *Scutellariae radix* (Huang-Qin), and *Glycyrrhizae Radix* et *Rhizoma Praeparata cum Melle* (Gan-Cao), was treated for “Xiaoke,” namely T2DM. GQD treatment conspicuously modulated gut microbial metabolism by degradation of choline into methylamines, together with a decrease in FBG and an expansion of islets in STZ and high-fat-diet-induced diabetic rats (Tian S. et al., 2013). In addition, GQD could modulate the composition of the intestinal microbiota during T2DM clinical treatment, especially enrich the quantity of beneficial bacteria such as *Faecalibacterium* spp., and it was found that structural alterations in the gut microbiota were associated with the anti-diabetic effects of GQD (Xu et al., 2015). What’s more, in the study of Wang et al. (2016), the quality and quantity of *Lactobacillus* and *Bacteroides* genus were significantly increased with the increasing concentration of POCS. The results indicated that POCS could restore the intestinal microbiota balance for the treatment of T2DM. Because most of the herbal drugs were usually orally administrated, the modulation of the herbal drugs on the intestine microbiota has been new mechanistic understanding of the natural products in DM treatment.

Compared with initial time-consuming laboratory experiments, network pharmacology approaches for well-known pathways with various natural products will be more efficient for antidiabetic drug discovery. Based on combining the network, chemical, pharmacological, biomedical and computational results, we can achieve multi-components and multi-targets therapy for DM.

## Applications of Network Pharmacology for DM Therapy

Network pharmacology, as a powerful tool to elucidate complex relationships from a systematic and biological level, has been more and more popular in recent years. It has been applied to various complex chronic diseases, such as cardio-cerebral ischemic diseases (Gu et al., 2013b), cancer (Azmi et al., 2010; Zhang et al., 2012; Poornima et al., 2016), pancreatitis (Hong et al., 2016), gout (Zhao et al., 2015), Alzheimer’s disease and DM and other metabolic disease (Loscalzo, 2011). For DM therapy, network pharmacology has been widely used in the mechanism studies.

According to the study of Liu et al. (2007), a gene network enrichment analysis (GNEA) was used to identify two sets of genes, IS-HD and NR-HD gene sets, which was associated with insulin signal and a network of nuclear receptors. They identified a network of PPI between members from IS-HD and NR-HD gene sets that might facilitate signal. By using GNEA, it was found that insulin-signal process was obviously transcriptionally altered in IR and T2DM. Their results also indicated that integrating high-throughput microarray studies together with PPI networks was an effective method to prove the existing biological processes associated with a complex disorder (Liu et al., 2007).

In the study of Li et al. (2014), the network pharmacology method was used to assist detecting the potential antidiabetic ingredients from the GQD. They collected 287 available chemical components, with 42 of them found in Ge-Gen, 57 of them found in Huang-Qin, 22 of them found in Huang-Lian, and 166 of them found in Gan-Cao. In this study, network target analysis was applied to elucidate the relationships between herbal ingredients and bioactivities. And the network pharmacology method also contributed to better understand the action mechanisms of GQD formula. It was also found that 4-Hydroxymephenytoin, a novel compound from *Puerariae Lobatae radix* (Ge-Gen), could increase the insulin secretion and improve IR (Li et al., 2014). The network pharmacology approach had been an effective strategy for the modern research on herbal drugs on the DM treatment, and the multi-active compounds could be identified and the multi-targets action mechanisms could be elucidated.

As the second most consumed beverage in the world next to water, Tea (*Camellia sinensis L.*) had been used as antidiabetic candidate for a long time according to folk remedies (Chen et al., 2007). Tea polyphenols (GTPs) was one of the main components of tea, mainly including EGCG, EGC, ECG, and EC. Different GTPs could act on different targets in the signal network of complex diseases (diabetes, cancer, cardiovascular, etc.), resulting in a synergistic effect, which was in accordance with the theory of network pharmacology. Study of network pharmacology approach on GTPs identified three pathways, p53 signal pathway, neurotrophin signal pathway, and T2DM pathway, which related to DM from all 147 pathways. And they established a diabetes-related pathway by integrating these three pathways (Zhang S. et al., 2014).

In order to find the potential inhibitors from TCM for the T2DM-related targets, Tian S. et al. (2013) developed an integrated approach that combined molecular docking and pharmacophore mapping, and they established the compound-target interaction network. A total of 2,479 non-duplicated compounds from these 32 Chinese herbs of 52 classical TCM formulas were found from two databases. The Bayesian classifiers with satisfactory discrimination capabilities for 15 targets were employed to screen the 2,479 compounds. The results showed that molecular docking or pharmacophore mapping could give satisfactory predictions for most targets. It was proved that some herbal ingredients could directly interact with T2DM related targets, while some ingredients relieved T2DM via antioxidant effects or other supplementary pharmacological effects (Tian S. et al., 2013).

Zhao H.L. et al. (2012) reported that male Zucker diabetic fatty rats were treated by JCU, a kind of TCM, which contained three herbs and berberine. The results showed that JCU could improve hyperglycemia, ameliorate IR, normalize liver enzyme and hepatocyte ballooning degeneration. On three levels including miRNA level, protein level and mRNA level, the study elucidated the action mechanisms of JCU, regulating the critical cytokines on the related pathways including improving the quality and quantity of cell survival, increasing glucose uptake, and ameliorating insulin sensitivity (Zhao H.L. et al., 2012). It in-depth validated that there was of great significance of multi-components TCM to treat DM.

In the work of Gu et al. (2011), the combination of molecular docking and network pharmacology analysis was developed to illustrate the action mechanism of Tangminling Pills for T2DM therapy. The drug–drug network and drug-target network illustrated that more than 100 active compounds of 667 compounds in the formula would target 37 target proteins related to T2DM. That is, they played important roles in the biological network. As well as the critical ingredients in Tangminling Pills were predicted and some of them had been reported in literatures. In addition, several compounds including Rheidin A, Sennoside C, Rheidin C, procyanidin C1, and Dihydrobaicalin were of importance as the antidiabetic candidate due to its pharmacological effects (Gu et al., 2011).

## Conclusion and Perspectives

Diabetes mellitus, a complicated metabolic disease, is a currently health problem causing significant mobility and mortality worldwide, which can cause various complications such as kidneys dysfunction, eyes damage, and cardiovascular diseases. Current antidiabetic drugs are still limited due to single-compound, single-target, side-effects, drug tolerance and low efficacy and they can’t cure DM completely. And it is significant that the world populations are heterogeneous and genetic polymorphisms in pharmacologically relevant genes varying across geographical region (Suarez-Kurtz et al., 2014). As a novel perspective, network pharmacology is becoming more and more popular in drug discovery. Compared with the single target of the current antidiabetic drugs, it is an promising approach which can combat the complex problems (Tao et al., 2014). It may also contribute to better explain the low clinical efficacy, side effects and toxicity of the current clinical drugs. At the same time, natural products have vast structural diversity and various bioactivities, which can provide the opportunities to find different lead compounds, different targets for DM. Hence, limited studies result in an urgent need to develop novel network pharmacology approaches to change current theories of drug discovery and to improve our understanding of action mechanisms. Although network pharmacology of natural products is still in its infancy, the appropriate use of network pharmacology approaches may initiate new directions, overcome the disadvantages of current antidiabetic therapies as well as contribute new insights into the discovery of novel antidiabetic drugs. Specific researches can be done to better elucidate the usage of natural products, and explore the possible action mechanisms.

## Author Contributions

WL drafted and revised the manuscript. GY, YP, and CW helped in literature survey and manuscript preparation. HC contributed to the conception and design of the work, revised and improved the manuscript. All authors approved on the finally submitted version of the manuscript.

## Conflict of Interest Statement

The authors declare that the research was conducted in the absence of any commercial or financial relationships that could be construed as a potential conflict of interest.

## Footnotes

1 http://www.diabetesatlas.org

2 http://www.who.int/en/

3 http://bioinformatics.charite.de/supernatural

4 http://www.ebi.ac.uk/chebi/

5 http://dnp.chemnetbase.com
